# Supplementary material for: Two-Stage Bio-Hydrogen and Polyhydroxyalkanoate Production: Upcycling of Spent Coffee Grounds
Source: Polymers (Basel). 2023 Jan 29;15(3):681. doi: 10.3390/polym15030681 (PMC9919241; doi:10.3390/polym15030681)
Supplement: Supplementary file 1 [file polymers-15-00681-s001.zip › polymers-2126948-supplementary.pdf]

Article

Two-stage Bio-hydrogen and Polyhydroxyalkanoate Production: Upcycling of Spent Coffee Grounds

Beom-Jung Kang [1,†], Jong-Min Jeon [1,†], Shashi Kant Bhatia [2], Do-Hyung Kim [3], Yung-Hun Yang [2], Sangwon Jung [4] and Jeong-Jun Yoon [1,*]

[1] Green & Sustainable Materials R&D Department, Korea Institute of Industrial Technology (KITECH), Chunan-si 31056, Republic of Korea
[2] Department of Biological Engineering, Konkuk University, Seoul 27478, Republic of Korea
[3] Sustainable Technology and Wellness R&D Group, Korea Institute of Industrial Technology (KITECH), Jeju-si 63243, Republic of Korea
[4] Department of Bio and Fermentation Convergence Technology, Kookmin University, Seoul 02707, Republic of Korea
* Correspondence: jjyoon@kitech.re.kr; Tel.: +82-41-589-8266
† These authors contributed equally to this work.

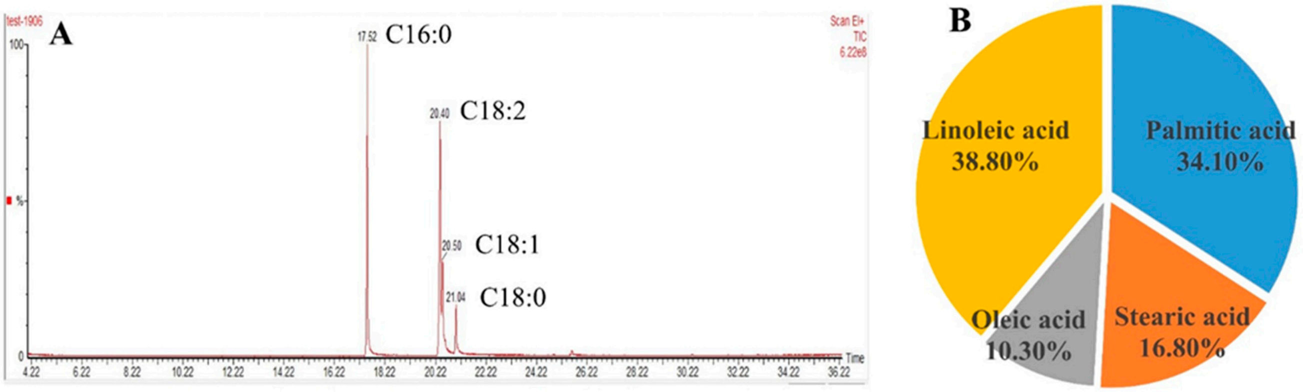

Figure S1. Qualitative and quantitative analysis of spent coffee grounds oil (A) GC/MS graph of qualitative analysis (B) composition of fatty acids in SCGs oil.

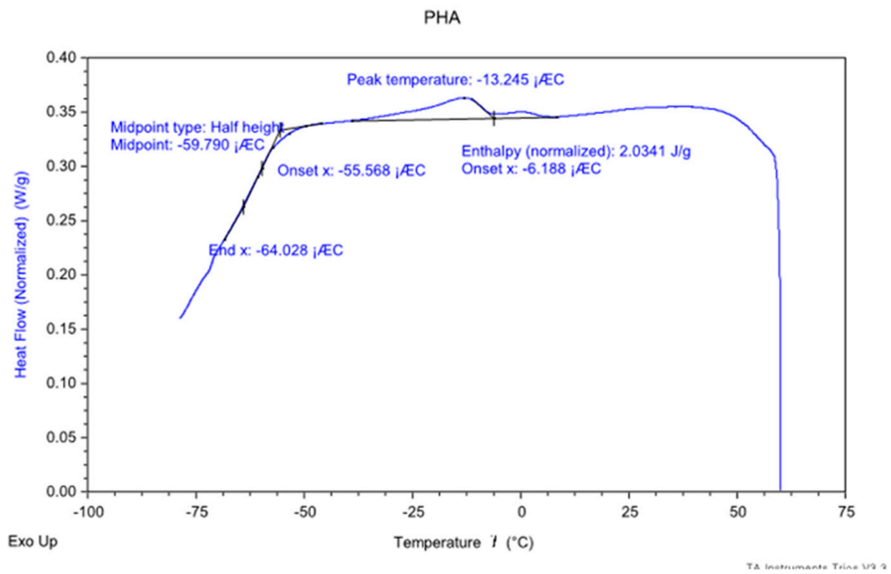

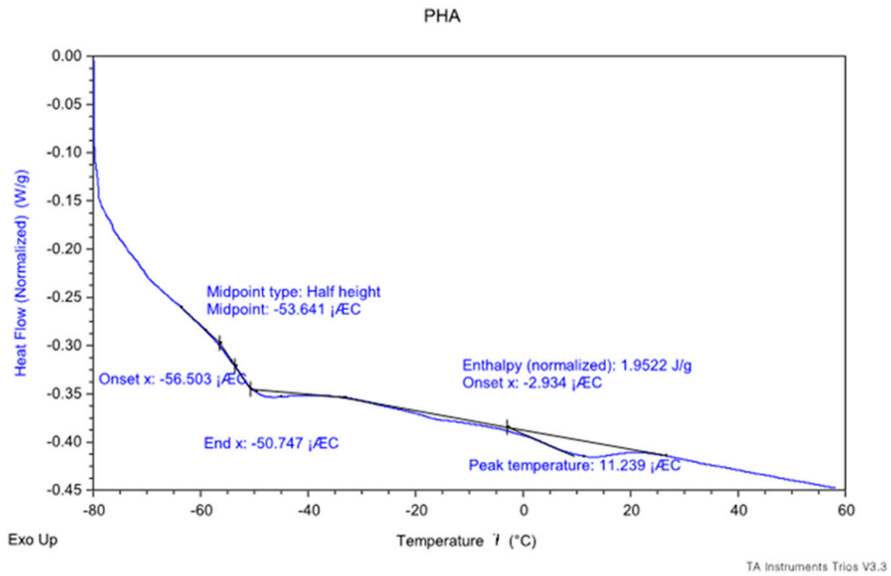

Figure S2. Differential scanning calorimetry results of mcl-PHA.

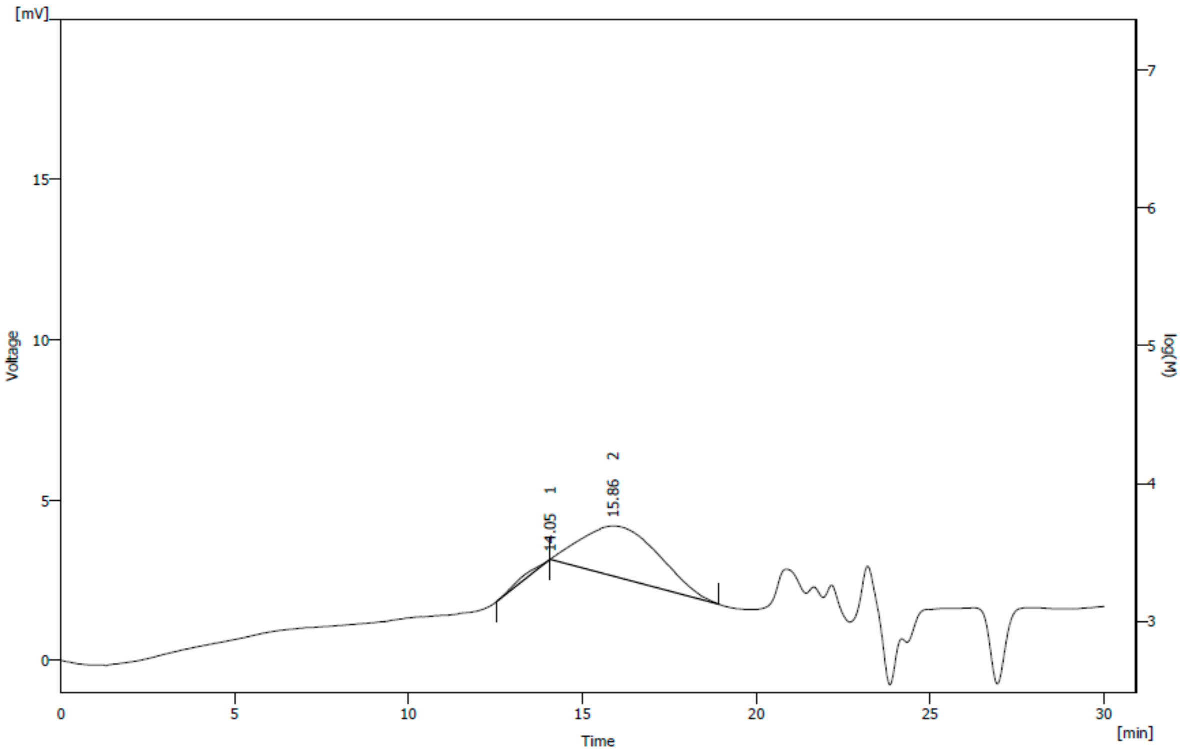

Figure S3. Gel permeation chromatography result of mcl-PHA.
